# Bilobalide Induces Apoptosis in 3T3-L1 Mature Adipocytes through ROS-Mediated Mitochondria Pathway

**DOI:** 10.3390/molecules28176410

**Published:** 2023-09-02

**Authors:** Su Bu, Anran Xiong, Zhiying Yang, Faycal Aissa-Brahim, Ying Chen, Yichun Zhang, Xunyong Zhou, Fuliang Cao

**Affiliations:** 1 College of Biology and the Environment, Nanjing Forestry University, Nanjing 210037, China; narnagx@163.com (A.X.); zhiying@njfu.edu.cn (Z.Y.); faycalab@outlook.com (F.A.-B.); chynjfu@163.com (Y.C.); 2 College of Forestry, Nanjing Forestry University, Nanjing 210037, China; yichun.zhang09@gmail.com; 3 HC Enzyme (Shenzhen) Biotech Co., Ltd., Shenzhen 518001, China; zimmenchow@126.com; 4 Co-Innovation Center for Sustainable Forestry in Southern China, Nanjing Forestry University, Nanjing 210037, China

**Keywords:** bilobalide, mature adipocyte, apoptosis, reactive oxygen species (ROS)

## Abstract

Bilobalide exhibits numerous beneficial bioactivities, including neuroprotective, anti-inflammatory, and antioxidant activity. Our previous study demonstrated that bilobalide inhibits adipogenesis and promotes lipolysis. The dose-dependent cytotoxicity was found to be specific to the mature adipocytes only, indicating the potential for regulating apoptosis in them. Herein, we aimed to investigate the apoptotic effects of bilobalide on 3T3-L1 mature adipocytes and elucidate the underlying mechanisms thereof. Flow cytometry analysis (FACS) revealed the pro-apoptotic effects of bilobalide on these cells. Bilobalide induced early apoptosis by reducing the mitochondrial membrane potential (MMP). DNA fragmentation was confirmed using TUNEL staining. Additionally, bilobalide increased the intracellular reactive oxygen species (ROS) levels and activities of Caspases 3/9. Pre-treatment with NAC (an ROS scavenger) confirmed the role of ROS in inducing apoptosis. Moreover, bilobalide up- and down-regulated the expression of Bax and Bcl-2, respectively, at the mRNA and protein expression levels; upregulated the Bax/Bcl-2 ratio; triggered the release of cytochrome c from the mitochondria; and increased the protein expression of cleaved Caspase 3, cleaved Caspase 9, and PARP cleavage. These results support the conclusion that bilobalide induces apoptosis in mature 3T3-L1 adipocytes through the ROS-mediated mitochondrial pathway, and offers potential novel treatment for obesity.

## 1. Introduction

Obesity is a global health problem due to its association with diverse diseases, including type 2 diabetes and cardiovascular and inflammatory disorders, as well as several types of cancer [1,2,3]. Energy impairment between storage and expenditure leads to overweight and excessive fat mass accumulation; this is an outcome of the processes that control the number and size of adipocytes, including proliferation, adipogenesis, and lipolysis. Many studies have focused on blocking adipogenesis to prevent the expansion of adipose tissue [4,5,6,7]. Evidence from several in vivo and in vitro studies have indicated that apoptosis is a significant factor in adipocyte depletion during weight reduction [8,9,10,11,12]. In response to well-studied stimuli, such as leptin or tumor necrosis factor alpha (TNF-α), adipose tissue can undergo phenotypic modifications to adapt apoptosis and modulate body fat mass reduction [13,14].

Apoptosis is a form of programmed cell death that results in the removal of cells with minimal inflammation. Therefore, decreasing the number of white adipocytes by apoptosis could be a novel strategy for combating obesity and its associated metabolic disorders. In this context, upregulation of fat mass and obesity-associated gene (FTO), one of the hallmarks of adipocyte metabolism, has been shown to attenuate the expression of pro-apoptotic factors, including Caspases 3 and 9, and Bax in vivo and in vitro; the upregulation of FTO also inhibited mitochondria-dependent apoptosis [15]. FOXO1, a downstream target of the PI3K/Akt signaling pathway, is highly involved in 3T3-L1 adipocyte apoptosis. Phosphorylation of FOXO1 elicits cell cycle arrest at multiple checkpoints represented by G1/S and G0/G1 transition in 3T3-L1 cells and stellate hepatocytes, respectively [16]. Other studies have revealed that phosphatidylcholine induces fat melting, apoptosis, and lipolysis in a dose-dependent manner via the TNF-α pathway. Phosphatidylcholine potentiates the transcriptional activity of TNF-α through the direct binding of NFκB1 and NFκB2 to their promoter, and also increases Caspase 3 activity [17,18].

Bilobalide, a sesquiterpene lactone, is one of the principal constituents of *Ginkgo biloba* leaves. It has been evaluated on neuronal, cancer, and fat cells for its beneficial properties, namely the abrogation of inflammation, oxidative stress, and hypoxia. Nevertheless, the underlying mechanisms are highly tissue-dependent and not yet fully elucidated [19]. It has been reported that bilobalide exerts pro-survival signals through the activation of the PI3K/Akt pathway in neuroblastoma cell lines, and alleviates hydrogen-peroxide-induced oxidative stress in melanocytes [20,21]. Moreover, bilobalide played a positive role in the treatment of Parkinson’s disease by inhibiting the apoptosis of SH-SY5Y cells ensuing from the damage caused by oxygen–glucose deprivation; bilobalide also ameliorated the apoptosis of nerve cells in α-synaptic nucleoprotein aggregation [22]. Bilobalide exhibits a therapeutic effect on gastric cancer by inhibiting the proliferation of gastric carcinoma cells and by inducing their apoptosis instead [23]. Similarly, bilobalide inhibits the proliferation of vascular smooth muscle cells and induces apoptosis in them, thereby reducing inflammation and improving atherosclerosis in mice [24].

We previously revealed that bilobalide suppresses adipogenesis in 3T3-L1 preadipocytes and promotes lipolysis through AMPK activation. Interestingly, a relatively greater cytotoxicity was observed in mature adipocytes [25]. Compared to other cell types, mature adipocytes are resistant to apoptosis. However, whether or not the enhanced reduction in cell viability by bilobalide was due to apoptosis has not yet been understood. In this study, we aimed to evaluate the apoptotic effects of bilobalide on 3T3-L1 mature adipocytes and the underlying mechanisms thereof.

## 2. Results

### 2.1. Bilobalide Induces Apoptosis in 3T3-L1 Adipocytes

**Figure 1:**
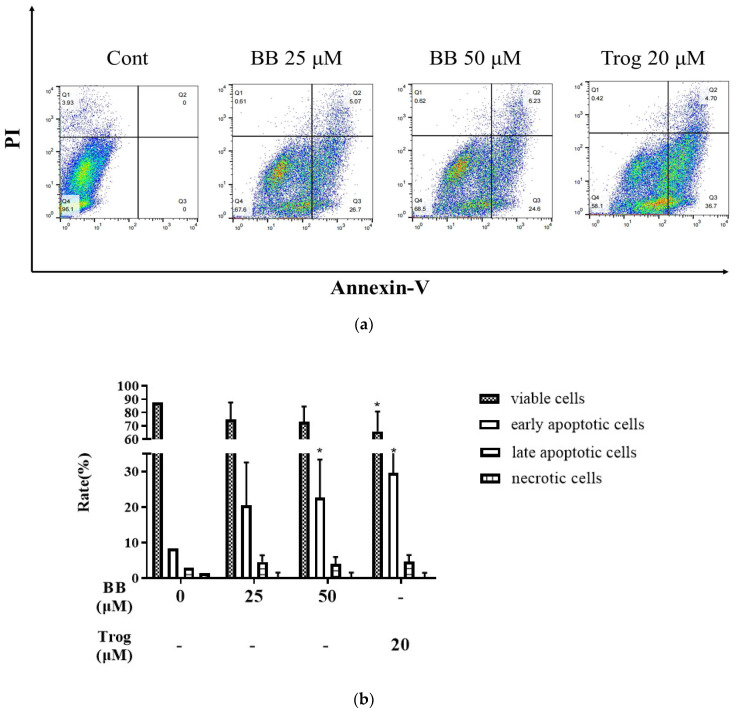
Bilobalide (BB) induced apoptosis in 3T3-L1 mature adipocytes. Apoptosis was assessed by flow cytometry using mature 3T3-L1 adipocytes, treated with different concentrations of bilobalide (0, 25, 50, 100 μM) for 48 h: (**a**) cell apoptosis rate was assessed through flow cytometry. The results shown here are representative from three independent experiments; (**b**) the histograms are presented as mean ± SD. All experiments were repeated three times independently with 3 replicates for each experiment. Each value was normalized to control, and the control was set to 100%. *, represent *p* < 0.05, vs. control.

Apoptosis is programmed cell death with characteristic changes, including nuclear fragmentation and cell shrinkage. We investigated whether the reduction in 3T3-L1 adipocyte numbers is due to bilobalide-induced apoptosis. The apoptosis rate of 3T3-L1 adipocytes after 48 h of treatment with different concentrations of bilobalide was analyzed using Annexin V-488 and PI double-stained flow cytometry. The results are shown in Figure 1. Compared to the no-treatment control, the proportion of viable cells treated with bilobalide decreased from 87.43% to 74.83%, 73.19%, and 65.77%, respectively, although insignificantly. Among the 3T3-L1 mature adipocytes treated with 25 μM, 50 μM bilobalide, and 20 μM thiazolidinediones (Trog), the proportion of early apoptotic cells increased significantly from 8.26% to 20.52%, 22.67% (*p* < 0.05), and 29.61% (*p* < 0.05), respectively, suggesting that bilobalide can induce early apoptosis in 3T3-L1 adipocytes. Compared to the control group, the proportion of late-apoptotic cells upon bilobalide (25 and 50 μM) and Trog (20 μM) treatment also increased from 2.87% to 4.52%, 4.00%, and 4.57%, respectively, although without significant differences. The proportion of necrotic cells in groups of the control, bilobalide (25, 50 μM), and Trog (20 μM) treatment was 1.44%, 0.14%, 0.15%, and 0.06%, respectively, without significant differences. These results verified that bilobalide induces the apoptosis of 3T3-L1 mature adipocytes by promoting early apoptosis in cells.

**Figure 2:**
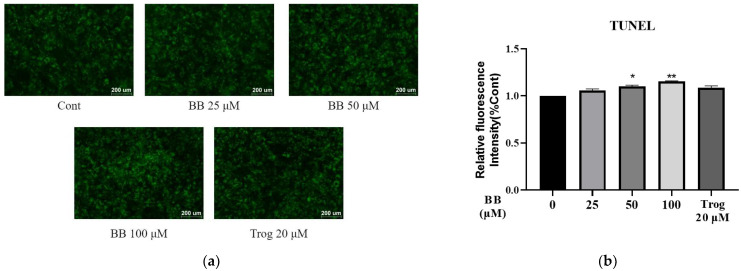
TUNEL assay was performed in mature adipocytes treated with bilobalide (BB) for 48 h: (**a**) the picture captured by a fluorescence microscope (100× magnification) represents the intracellular TUNEL level. The fluorescent photographs shown here are representative of randomly selected areas; (**b**) the fluorescence intensity was quantified by Image J software, version 1.53c The histograms are presented as mean ± SD. All experiments were performed three times independently with three randomly selected fields. Each value was normalized to control, and the control was set to 100%. *, and ** represent *p* < 0.05, and *p* < 0.01, respectively, vs. control.

We then performed a TUNEL assay to evaluate the apoptotic effect of bilobalide. With the dose of bilobalide elevated from 0 μM, 25 μM, and 50 μM to 100 μM, we observed a slight but steady increase in the relative fluorescence intensity in a dose-dependent manner (Figure 2). The 50 μM and 100 μM treatment groups exhibited significantly higher fluorescence intensity (1.1 and 1.2 times that of the control group, respectively; *p* < 0.05), indicating that bilobalide promotes the apoptosis of 3T3-L1 adipocytes.

### 2.2. Effect of Bilobalide on Reactive Oxygen Species (ROS) Generation in Adipocytes

**Figure 3:**
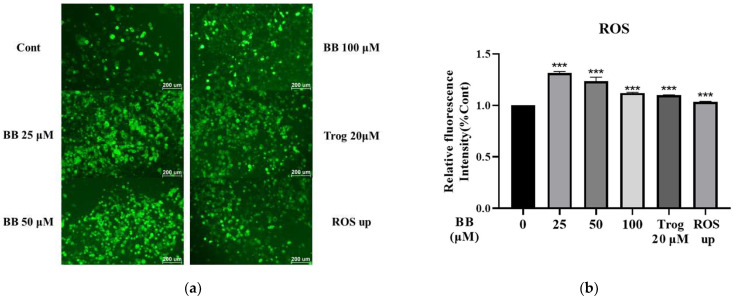
Effect of bilobalide (BB) on ROS generation in 3T3-L1 mature adipocytes: (**a**) fluorescence microscopy (100× magnification) was used to observe the changes in the level of intracellular ROS generation; (**b**) relative fluorescence intensity was detected using a fluorescence microplate reader. The relative change ratio of fluorescence intensity in the treatment group compared to the control group represents the relative fluorescence intensity. The values are presented as means ± SD from three independent experiments with three randomly selected fields for each experiment. Each value was normalized to control, and the control was set to 100%. *** represents *p* < 0.001, vs. control.

There are several reports suggesting that apoptosis in cells is usually triggered by the generation of ROS. We examined the effects of bilobalide on ROS generation in 3T3-L1 adipocytes. The fluorescence intensity of ROS in 3T3-L1 mature adipocytes treated with bilobalide for 16 h was observed by fluorescence microscopy. Representative images are shown in Figure 3a.

In addition, the fluorescence intensity was also measured using a Multi-Mode Microplate Reader. As shown in Figure 3b, compared to the control group, the ROS levels increased significantly at all concentrations, indicating that bilobalide can increase the generation of ROS and promote cell apoptosis; the highest level of 1.32-fold was observed in the 25 μM bilobalide treatment group (*p* < 0.001).

**Figure 4:**
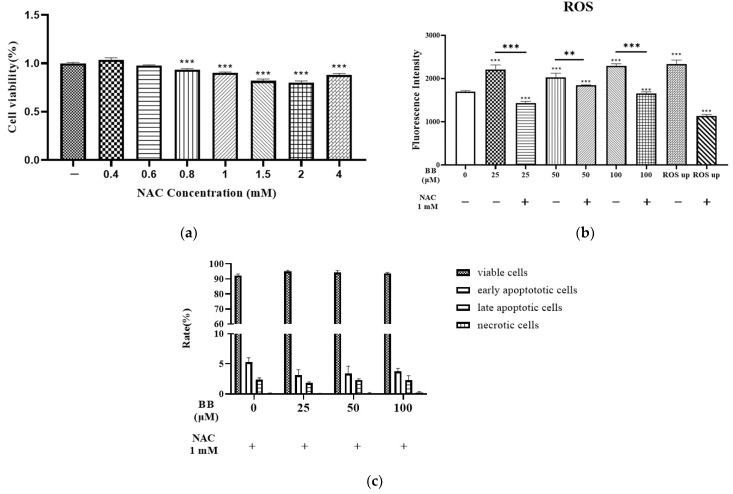
ROS generation and flow cytometry results of bilobalide (BB) treatment with or without NAC: (**a**) MTT assay for NAC; (**b**) intracellular ROS levels were determined after treatment with bilobalide in combination with NAC (1 mM); (**c**) apoptosis rate of mature adipocytes upon treatment with different concentrations of bilobalide in combination with NAC (1 mM). The values are presented as mean ± SD from three independent experiments with three replicates for each experiment. Each value was normalized to control, and the control was set to 100%. **, and *** represent *p* < 0.05, *p* < 0.01, and *p* < 0.001, respectively, vs. control.

To further confirm whether the apoptosis induced by bilobalide was mediated by ROS, intracellular ROS levels in 3T3-L1 mature adipocytes were detected after 30 min treatment with bilobalide alone or in combination with 1 mM NAC (an ROS scavenger). The MTT assay revealed that the cytotoxicity of NAC was very low up to a 1 mM concentration (Figure 4a). While the increase in ROS production due to bilobalide was significantly reduced by NAC in all the treatment groups (Figure 4b), the apoptotic rate showed no change in comparison with the control group when treated with different concentrations of bilobalide in combination with NAC (Figure 4c). These results indicate that apoptosis induced by bilobalide was mediated by ROS.

### 2.3. Bilobalide Treatment Reduced Mitochondrial Membrane Potential (MMP) in 3T3-L1 Mature Adipocytes

Mitochondria are regulatory centers and an important site of apoptosis. A change in MMP is an important factor in causing apoptosis. A reduction in MMP is the earliest event in the process of apoptosis cascade. It causes a series of biochemical changes in the mitochondrial membrane, leading to a cellular apoptotic cascade.

**Figure 5:**
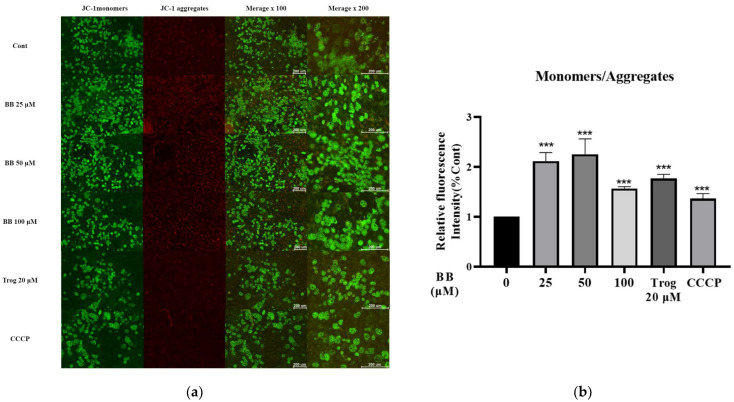
Effect of bilobalide (BB) on the mitochondrial membrane potential (MMP) of 3T3-L1 mature adipocytes: (**a**) fluorescence microscopy was used to observe the changes in intracellular MMP. The ratios of green /red fluorescence were calculated to characterize MMP. The representative images of JC-1 stained 3T3-L1 mature adipocyte were presented; (**b**) statistical analysis of fluorescence semi-quantification was performed on three independent experiments with three randomly selected fields for each experiment. Scale bar is shown as 200 μm in both 100× and 200× merged images. The values are presented as mean ± SD. Each value was normalized to control, and the control was set to 100%. *** represent *p* < 0.001 vs. control.

To further investigate the molecular mechanism of bilobalide-induced apoptosis, we examined the effect of bilobalide on mitochondrial function in 3T3-L1 mature adipocytes. MMP was detected using the Enhanced Mitochondrial Membrane Potential Assay Kit with JC-1 dye. As shown in Figure 5, compared to the control group, the fold changes in the ratio of JC-1 monomer/JC-1 aggregate upon treatment with 25, 50, and 100 μM bilobalide, 20 μM Trog, and 10 μM CCCP were 2.11—(*p* < 0.001), 2.25—(*p* < 0.001), 1.57—(*p* < 0.001), 1.77—(*p* < 0.001), and 1.37—(*p* < 0.001)-fold, respectively. The data indicated that bilobalide reduced the MMP of 3T3-L1 adipocytes, suggesting that bilobalide could effectively promote apoptosis through the mitochondrial pathway.

### 2.4. Effects of Bilobalide on Caspase 3/9/8 Activities in Apoptosis

**Figure 6:**
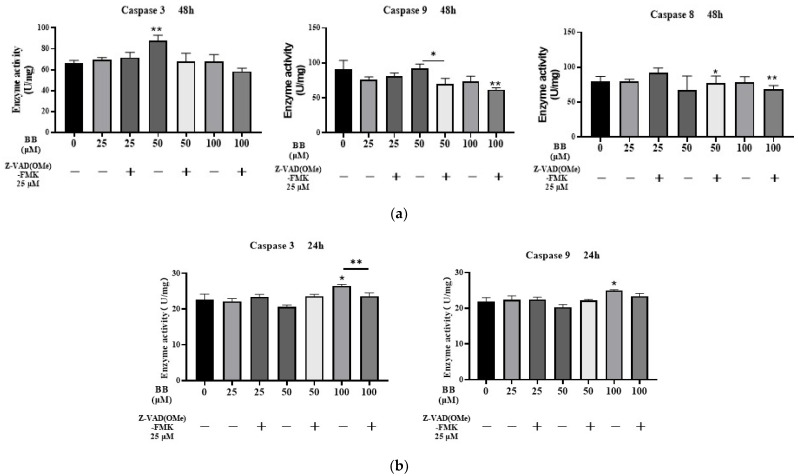
Effect of bilobalide (BB) on Caspase 3/9/8 activity in 3T3-L1 adipocytes: (**a**) the enzyme activities of Caspase 3/9/8 in mature adipocytes treated with different concentrations of bilobalide for 48 h; (**b**) the enzyme activities of Caspase 3/9 in mature adipocytes treated with different concentrations of bilobalide for 24 h. The values are presented as mean ± SD from three independent experiments with three replicates for each experiment. Each value was normalized to control, and the control was set to 100%. * and ** represent *p* < 0.05, and *p* < 0.01, respectively, vs. control.

Caspases (cysteinyl aspartic acid proteases) are a family of proteases that can destroy important intracellular proteins and trigger apoptosis. The activities of Caspase 3, Caspase 9, and Caspase 8 were measured after 48 h of treatment with different concentrations of bilobalide (25, 50, and 100 μM). Compared to the control group, the 50 μM bilobalide treatment resulted in a significant increase in Caspase 3 activity (*p* < 0.01); after a 30-min pre-treatment with a caspase pan-inhibitor—Z-VAD(OMe)-FMK—the Caspase 3 activity in the 50 μM bilobalide treatment group was found to be significantly reduced (*p* < 0.05). Caspase 9 activity increased slightly upon 50 μM bilobalide treatment, but was abrogated by Z-VAD(OMe)-FMK treatment (*p* < 0.05). However, no significant changes were observed in Caspase 8 activity in cells treated with bilobalide alone at any concentration. But when the cells were treated with the combination of the inhibitor and 50 and 100 μM bilobalide, the activity of Caspase 8 was decreased slightly in the 50 μM and 100 μM bilobalide treatment group (*p* < 0.05 and *p* < 0.01, Figure 6a).

To determine the most sensitive time point of the caspase response to bilobalide, we collected adipocytes treated with bilobalide for 24 h and assayed the activities of Caspase 3 and Caspase 9. To further investigate the effect of bilobalide on Caspase 3/9 activity in a shorter time, all bilobalide treatments were combined with a 30-min pre-treatment with Z-VAD(OMe)-FMK. As shown in Figure 6b, the activities of Caspase 3 and Caspase 9 were significantly increased after 100 μM bilobalide treatment (*p* < 0.05). Notably, we found that Z-VAD(OMe)-FMK significantly decreased the activity of Caspase 3 induced by 100 μM bilobalide (*p* < 0.001).

In summary, our results indicate that bilobalide enhances the activities of Caspase 3 and Caspase 9 in 3T3-L1 mature adipocytes, but had no effect on Caspase 8 activity. Pre-treatment with Z-VAD(OMe)-FMK effectively reversed the activation effect of bilobalide on Caspase 3 (*p* < 0.05). In the apoptotic pathways, Caspase 8 serves as the initiator of the exogenous pathway of apoptosis, while Caspase 9 initiates the endogenous pathway. Overall, the Caspase 3/8/9 activity tests showed that bilobalide promoted an increase in the activity of Caspase 3 and 9 in 3T3-L1 mature adipocytes, suggesting that apoptosis induced by bilobalide was via the endogenous pathway.

### 2.5. The Effect of Bilobalide on the Expression of Apoptotic Genes in Adipocytes

**Figure 7:**
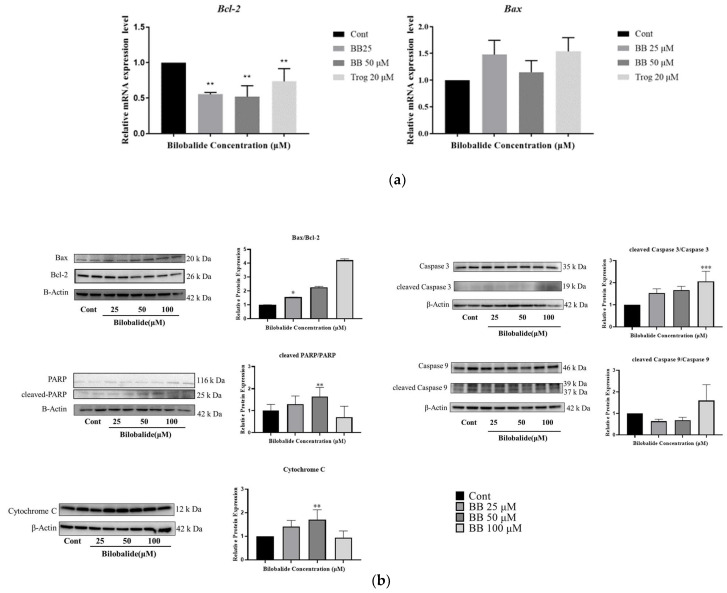
Bilobalide regulates apoptosis-related gene expression in 3T3-L1 adipocytes. Mature 3T3-L1 adipocytes treated with different concentrations of bilobalide (0, 25, 50, 100 μM) for 48 h: (**a**) relative mRNA expression of Bcl-2 and Bax was detected by qRT-PCR; (**b**) Western blot was used to detect the apoptotic proteins. β-Actin was employed as the loading control. The intensity of each band was quantified by densitometry analysis. The values are presented as mean ± SD from three independent experiments. Each value was normalized to control, and the control was set to 100%. *, **, and *** represent *p* < 0.05, *p* < 0.01, and *p* < 0.001, respectively, vs. control.

The relative mRNA expression levels of apoptotic genes in 3T3-L1 adipocytes treated with bilobalide were quantified using quantitative real-time polymerase chain reaction (qRT-PCR). As shown in Figure 7a, bilobalide significantly promoted apoptosis in 3T3-L1 adipocytes by increasing the expression of the pro-apoptotic gene *Bax* and abolishing the mRNA expression of the anti-apoptotic gene *Bcl-2* at 25μM and 50 μM concentrations. The transcriptional levels of *Bax* in the 25 μM and 50 μM treatment group were 1.61 and 1.23 times that of the control group (*p* < 0.01), respectively; and the transcriptional levels of *Bcl* were 0.55 and 0.52 times that of the control group (*p* < 0.01), respectively. The 20 μM Trog treatment group was included as the positive group.

We further examined the relative protein expression levels of apoptotic proteins, such as Bax, Bcl-2, cleaved poly (ADP-ribose) polymerase (PARP) and PARP, cleaved Caspase 3 and Caspase 3, cleaved Caspase 9 and Caspase 9, and cytochrome c, in 3T3-L1 adipocytes treated with bilobalide by Western blotting. As shown in Figure 7b, bilobalide clearly upregulated Bax expression and downregulated Bcl-2 expression. Thus, the Bax/Bcl-2 ratio substantially increased with the values of 1.56, 2.26, and 4.23, respectively, for the 25 μM, 50 μM, and 100 μM treatment groups (*p* < 0.05). Meanwhile, we calculated the ratio of cleaved protein level to the total protein expression level for PARP, Caspase 3, and Caspase 9, and then normalized them to the control group. The normalized ratio of cleaved PARP/PARP was significantly increased in the 50 μM treatment group compared with the control group (1.87 vs. 1, *p* < 0.05), while the normalized ratio of cleaved Caspase 3/Caspase 3 was elevated dramatically in the 100 μM treatment group (1.91 vs. 1, *p* < 0.001). We also saw an increasing trend in the normalized ratios of cleaved PARP/PARP (1.30 for 25 μM) and cleaved Caspase 3/Cascade 3 (1.74 for 25 μM and 1.89 for 50 μM) in lower-dose groups. However, the increase was not statistically significant. We did not detect a significant elevation in the normalized ratio of cleaved Caspase 9/Caspase 9 in any bilobalide treatment group. In addition, treatment with 50 μM bilobalide significantly increased the expression of cytochrome C compared with the control group (1.67 vs. 1, *p* < 0.01) (see details in Supplementary Materials).

These results suggest that bilobalide induces Caspase-3-dependent apoptosis in 3T3-L1 adipocytes.

## 3. Discussion

The prevalence of obesity has increased significantly all over the world in the last 50 years. It is a strong risk factor for the development of type 2 diabetes, cardiovascular diseases, and certain types of cancer [26]. Excessive accumulation of fat in the body is the result of an increase in the size (hypertrophy) and number (hyperplasia) of adipocytes, along with the infiltration of immune cells in the adipose tissue [27,28]. The number of adipocytes that build up the adipose tissues is stable over a prolonged period of time, and mature adipocytes are relatively resistant to apoptosis [8,29]. One-third of the adipose tissue consists of mature adipocytes, and the remaining tissue includes heterogeneous cells, such as fibroblasts, mesenchymal stem cells (MSCs), endothelial progenitor cells (EPCs), stromal cells, macrophages, T regulatory cells, red blood cells, and preadipocytes at various developmental stages. Preadipocytes make up 15–50% of the adipose tissue [30]. Targeting adipocyte apoptosis to reduce the volume of adipose tissues by bioactive components [2,13,31,32,33,34], surgical injection [35], and instrument intervention [36] are considered as promising strategies for combating obesity and its associated metabolic disorders.

Our previous study indicated that bilobalide can inhibit adipogenesis and promote lipolysis via the AMPK pathway. Furthermore, it exhibited significantly higher cytotoxicity in mature adipocytes in a dose-dependent manner, but very low cytotoxicity in preadipocytes [4], indicating its potential in regulating apoptosis in mature adipocytes. In the present study, we investigated the effect of bilobalide on apoptosis in mature adipocytes and the underlying mechanisms thereof.

Both the extrinsic and intrinsic pathways of apoptosis can be activated in adipocytes by different types of cellular stresses [37,38]. In the extrinsic pathway, death receptors bind extracellular ligands and transmit death signals to the intracellular space via cytoplasmic death domains anchored to the cell membrane [39]. In contrast, the intrinsic pathway of apoptosis is initiated within the cell, triggered by the loss in MMP, formation of a mitochondrial permeability transition pore (MPT), and release of pro-apoptotic factors, such as cytochrome c [40]. Apoptosis activates effector Caspase 3 by cleaving Procaspase-9 and producing active Caspase 9 [41].

Our flow cytometry results reveal that bilobalide induced the apoptosis of mature 3T3-L1 adipocytes. TUNEL staining indicated that bilobalide induced fragmentation of genomic DNA in mature adipocytes. Moreover, JC-1 staining indicated that bilobalide reduced MMP in 3T3–L1 mature adipocytes. In addition, bilobalide upregulated the expression of Bax and downregulated the expression of Bcl-2 at both the mRNA and protein expression levels, along with the upregulation of the Bax/Bcl-2 ratio. Bilobalide also led to the release of cytochrome c from the mitochondria into the cytoplasm. Similarly, cleaved Caspase 3 and cleaved Caspase 9, as well as PARP cleavage by bilobalide, were observed. Furthermore, bilobalide promoted the accumulation of intracellular ROS.

Previous studies have shown that ROS play vital roles in the regulation of diverse functional pathways involved in apoptosis, ferroptosis, and autophagy [42,43,44]. Consistent with the results of previous studies [23,45,46,47], our results suggest that apoptosis induction in mature adipocytes treated with bilobalide is a function of enhanced ROS generation. Meanwhile, the promotion of intracellular ROS generation can be reversed by the use of NAC, an ROS scavenger. Bilobalide has been shown to promote mitochondrial biogenesis and reverse hypoxic impairment by downregulating HIF-1α and restoring energy catabolism and oxygen consumption in mature adipocytes to normal levels. This highlights its potential role in adipocyte activation and increased energy degradation [15,19].

Although the induction of adipocyte apoptosis seems to be of some concern with respect to an increase in inflammation, increase in blood lipid concentration, ectopic lipid storage, and other metabolic disorders [38,48,49], maintaining apoptosis at a low level in adipocytes, ADSCs, and other cells in fat transplants or in autologous lipofilling procedures is mandatory to increase graft survival [50]. Many studies have shown a generally low inflammation index following the induction of adipocyte apoptosis [51,52]. Collectively, these results suggest that maintaining adipocyte apoptosis at an appropriate level is critical for adipose tissue development and homeostasis.

## 4. Materials and Methods

### 4.1. Chemicals and Reagents

Dulbecco’s modified Eagle medium (DMEM, 500 mL), Dulbecco’s phosphate-buffered saline (DPBS, 500 mL), fetal bovine serum (FBS, 500 mL), 0.25% trypsin (100 mL), and penicillin–streptomycin solution (100 mL) were purchased from Gibco (Rockville, MD, USA). Insulin (5 mL), dexamethasone (DEX,), and 3-isobutyl-1-methylxanthine (IBMX, 100 mg) were obtained from Sigma-Aldrich (St. Louis, MO, USA).

3T3-L1 preadipocytes (SCSP-5038) were obtained from the National Collection of Authenticated Cell Cultures, Shanghai Institutes for Biological Sciences, Chinese Academy of Sciences (Shanghai, China). Bilobalide (HPLC ≥ 98%) was obtained from Yuanye Bio-Technology (Shanghai, China).

Primary antibodies specific for Anti-Bcl2, Anti-Bax, Anti-Cleaved PARP, Anti-Caspase 3, and Anti-Caspase 9 were acquired from Cell Signaling Technology (Danvers, USA). Anti-PARP was acquired from Abcam (Cambridge, UK). Anti-cytochrome c was acquired from Proteintech Group (Wuhan, China). Anti-β-Actin was obtained from Trans-Gen Biotech (Beijing, China). Goat anti-mouse IgG and goat anti-rabbit IgG secondary antibodies were obtained from Abmart (Shanghai, China).

One Step TUNEL Apoptosis Assay Kit, Reactive Oxygen Species Assay Kit, Enhanced Mitochondrial Membrane Potential Assay Kit with JC-1, Caspase 3 Activity Assay Kit, Caspase 9 Activity Assay Kit, and Caspase 8 Activity Assay Kit were purchased from Beyotime Biotechnology (Shanghai, China). The MiNiBEST Universal RNA Extraction Kit and SYBR Premix Ex Taq II Kit were from TaKaRa (Dalian, China). The Annexin V-FITC/PI Apoptosis Detection Kit was obtained from YEASEN (Shanghai, China). The molecular weight markers used for SDS-PAGE were Precision Plus Protein™ Dual Color Standards (10–250 kD), purchased from BioRad (Hercules, CA, USA).

### 4.2. Cell Culture

3T3-L1 preadipocytes cells have a fibroblast-like morphology and can be maintained in DMEM (1 g/mL glucose) with 10% FBS and 1% penicillin–streptomycin in a humidified atmosphere with 5% CO_2_ at 37 °C. For cell passaging, cells were first seeded at 3 × 10^3^ cells/cm^2^. When 80% confluence was reached, cells were washed with PBS 3 times followed by 0.25% trypsin treatment and 1–2 min of incubation in the cell incubator. Cells were then gently beaten. When most of the cells turned to round shape under the microscope, DMEM medium was added to terminate the trypsin digestion reaction. Cells were then washed by PBS, counted, and seeded for further passaging or differentiation. The initial passage number before experiments was P11.

For adipocyte differentiation, the preadipocytes were placed in growth medium (1 g/mL glucose DMEM, 10% FBS, 1% penicillin–streptomycin) when the cells reached confluence. Forty-eight hours post confluence, we initiated the adipocyte differentiation by replacing the growth medium with differentiation medium I (4.5 g/mL glucose DMEM, 10% FBS, 1% penicillin–streptomycin, 10 µg/mL insulin, 0.5 mM IBMX, and 1 µM DEX) and marked the day as day 0 of differentiation. Cells were cultured in differentiation medium I for 3 days. Thereafter, the medium was replaced with differentiation medium II (4.5 g/mL glucose DMEM, 10% FBS, 1% Pen-Strep, and 10 µg/mL insulin) and cells were cultured for 2 days, followed by maintenance in differentiation medium III (4.5 g/mL glucose DMEM, 10% FBS, 1% Pen-Strep. The differentiation medium III was refreshed every 2–3 days until the end of differentiation. The total differentiation time was 8–10 days unless otherwise mentioned in the specific experiments.

T75/T25 flasks and 6/12/24/96-well plates were used for the cell cultures according to the specific experimental design requirements.

### 4.3. Assessment of Apoptosis by Flow Cytometry

3T3-L1 mature adipocytes were differentiated until day 8, whereafter they were exposed to different concentrations of bilobalide (0, 25, 50, and 100 μM) for 48 h. Previous studies have shown that 20 μM thiazolidinediones (Trog) can induce apoptosis in 3T3-L1 adipocytes [53]; therefore, the 20 μM Trog treatment group was used as positive control for apoptosis in our experiment. The Annexin V-FITC/PI Apoptosis Detection Kit (YEASEN, Shanghai, China) was used to analyze the apoptosis rate according to the manufacturer’s protocol.

Briefly, at the end of the time point, cells were collected by trypsin digestion, washed with PBS, resuspended with 100 μL binding buffer, and filtered using a FALCON 100 μM cell strainer (Corning, NY, USA). Thereafter, 5 μL Annexin V-FITC and 10 μL PI staining solution were added in the filtration. The filtration samples were incubated in the dark on ice for 10–15 min, and then analyzed by a BD flow cytometer (San Jose, CA, USA), and the FACS files were analyzed using FlowJo software (version 10). In the 20 μM Trog treatment group, the cells were stained with PI and Annexin V-FITC only, respectively, and both PI and Annexin V-FITC, as well as no dye for the voltage setting. The viable cells were Annexin V−/PI−, early apoptotic cells were Annexin V+/PI−, late apoptotic cells were Annexin V+/PI+, and necrotic cells was Annexin V−/PI+.

### 4.4. Measurement of Intracellular ROS Generation

The Reactive Oxygen Species Assay Kit (Beyotime Biotechnology, Shanghai, China) was used for the assay of intracellular ROS generation. The determination of ROS generation was based on the oxidation of 2,7-dichlorodihydro-fluorescein diacetate (DCFH-DA) into a fluorescent dye, 2,7-dichlorodihydro-fluorescein (DCFH) by cellular oxidants. The fluorescence intensity is proportional to the level of peroxide produced by the cells. Mature 3T3-L1 adipocytes grown and differentiated from 3 × 10^4^ cells/well in 6-well plates were treated with different concentrations of bilobalide (0, 25, 50, and 100 μM) for 16 h. Cells treated with 20 μM of Trog for 16 h were used as positive control for apoptosis. Meanwhile the mature adipocytes treated with Rosup (a positive reference substance provided by the kit) for 30 min were also used as positive control for ROS detection. Thereafter, the diluted DCFH (10 μM) was added to the 6-well plates and incubated at 37 °C in 5% CO_2_ for 30 min. After staining, the cells were washed three times with serum-free medium, and images were acquired using an inverted fluorescence microscope (Ti2-U, Nikon, Tokyo, Japan) with excitation and emission wavelengths of 488 and 525 nm, respectively. In addition, the fluorescence of samples was monitored using a spectrophotometer at excitation and emission wavelengths of 480 and 525 nm, respectively, using a BioTek Multi-Mode Microplate Reader (Synergy2, Vineland, NJ, USA).

### 4.5. TUNEL Analysis

The One Step TUNEL Apoptosis Assay Kit (Beyotime Biotechnology, Shanghai, China) was used to detect breaks in nuclear DNA in apoptotic tissues or cells. The exposed 3′-OH of the broken genomic DNA can be catalyzed by terminal deoxynucleotidyl transferase (TdT) to bind to fluorescein (FITC)-labeled dUTP (fluorescein-dUTP), which can be detected by fluorescence microscopy or flow cytometry. We performed TUNEL assay according to the manufacturer’s protocol. Briefly, mature 3T3-L1 adipocytes were treated with different concentrations of bilobalide (0, 25, 50, and 100 μM) for 48 h, with the 20 μM Trog treatment group included as positive control for apoptosis. The cells were then fixed in 4% formaldehyde solution for 30 min followed by permeabilization with 0.3% Triton-X 100 in PBS at room temperature for 5 min. The cells were then washed twice with PBS and labeled with 50 μL of the configured TUNEL assay detection solution at 37 °C for 1 h in the dark. Images were obtained using an inverted fluorescence microscope (Ti2-U, Nikon, Japan) with excitation and emission wavelengths of 488 and 525 nm, respectively, and the fluorescence intensity was semi-quantified using Image J software version 1.53c.

### 4.6. Measurement of MMP

MMP was analyzed using Enhanced Mitochondrial Membrane Potential Assay Kit with JC-1 dye (Beyotime Biotechnology, Shanghai, China). JC-1 dye accumulates in functional mitochondria, wherein the MMP (ΔΨm) is normally high and forms aggregates that emit red fluorescence. When the mitochondrial membrane depolarizes, JC-1 dye exhibits potential-dependent accumulation in the mitochondria, indicated by a fluorescence emission shift from red (590 nm) to green (530 nm). The loss of MMP is indicated by a decrease in the mean ratio of red/green fluorescence intensity. We performed MMP measurement according to the manufacturer’s protocol. Briefly, mature adipocytes were treated with different concentrations of bilobalide (0, 25, 50, and 100 μM) for 16 h, and the 20 μM Trog treatment group was used as positive control for apoptosis. Adipocytes treated with 10 μM CCCP for 20 min were also included as the kit positive control. The cells were washed once with PBS and incubated in the JC-1 working solution for 20 min at 37 °C in a cell incubator. The cells were washed twice with JC-1 staining buffer and examined using fluorescence microscopy. The excitation and emission wavelengths were 490 and 530 nm, respectively, for the detection of JC-1 monomer (red), while the excitation and emission wavelengths were 525 and 590 nm, respectively, for the detection of JC-1 polymer (green). Three fields were randomly selected for calculation of the average fluorescence intensity and mitochondrial membrane potential was calculated by the ratio of green to red.

### 4.7. Quantification of the Expression of Apoptotic Genes in 3T3-L1 Adipocytes

For the analysis of the mRNA expression of target apoptosis genes in bilobalide-treated adipocytes, qRT-PCR was performed. Total RNA was extracted using the MiniBEST Universal RNA Extraction Kit (Takara, Japan), and the RNA (1 μg) was reverse-transcribed to cDNA using the PrimeScript^®^ 1st Strand cDNA Synthesis Kit (Takara, Japan) following the manufacturer’s instructions. The cDNA was then amplified by specific primers using TB Green^®^ Premix Ex Taq™ II (Tli RNaseH Plus) and ROX plus (Takara, China) on a StepOnePlus qPCR thermal cycler (Applied Biosystems, Forster City, CA, USA). qPCR reactions were run in triplicate for each sample, and the transcription levels of every gene were normalized to the level of GAPDH. Relative expression was determined using the 2^−∆∆CT^ method. Values are presented as fold changes compared to the control. The specific primers used in this study are listed in Table 1.

### 4.8. Western Blot Analysis

The mature 3T3-L1 adipocytes treated with different concentrations of bilobalide were washed with PBS, trypsinized, and collected by centrifugation at 12,000 rpm for 10 min at 4 °C after repeating the step three times. The cell pellets were resuspended in Biosharp RIPA Q lysis buffer (Hefei, China) supplemented with phosphatase and protease inhibitors and incubated on ice for 5 min, and then followed by an ultrasonic process. The ultrasonic process was conducted using an Ultrasonic Homogenizer JY92-IIN of Scinntz Biotechnology (Ningbo, China) with a power setting of 35 W. Each cycle involved sonication for 4 s followed by a 6 s interval, repeated a total of 6 times. Subsequently, the sample was placed on ice and incubated for 10 min to facilitate further lysis. Following the above sonication, the supernatant was collected by centrifugation at 12,000 rpm for 10 min at 4 °C and used as crude total protein. Protein concentrations were determined using a BCA Protein Assay kit (Beyotime Biotechnology, Shanghai, China). The denatured protein samples (40 μg) were separated by 4–12% SDS-PAGE and transferred onto a 0.45 μM PVDF membrane (Millipore, Boston, MA, USA). The membrane was blocked with 5% nonfat dry milk or 5% BSA according to the protocol associated with the respective primary antibody in TBST buffer (20 mM Tris, 166 mM NaCl, and 0.1% Tween 20, pH 7.5) at room temperature for 1 h and washed three times with TBST buffer prior to incubation with primary antibodies in TBST buffer at 4 °C overnight. After three washes in TBST, the membranes were incubated with the corresponding horseradish peroxidase-conjugated secondary antibodies for 1 h and washed three times with TBST. The dilutions of all primary antibodies and the secondary antibodies are 1:1000 and 1:5000, respectively. The antibodies’ sources and their manufacturers can be found in Section 4.1. Chemicals and Reagents. Immunoreactive proteins were visualized using Clarity Max Western ECL Substrate (Bio-Rad, Shanghai, China) on a Chemi Doc XRS Imaging system (Bio-Rad, Shanghai, China), and the signals were quantified by densitometry using Image Lab software, version 5.2.1 (Bio-Rad, Shanghai, China).

### 4.9. Caspase 3/9/8 Assay and Their Specific Inhibitor Activities

To detect the effect of bilobalide in combination with or without Z-VAD(OMe)-FMK (caspase pan-inhibitor) on caspase activity, mature adipocytes were pretreated with 25 μM Z-VAD(OMe)-FMK for 30 min. After removal of Z-VAD(OMe)-FMK, the adipocytes were cultured with bilobalide at different concentrations (0, 25, 50, and 100 µM) for 24 or 48 h. Cells were trypsinized, washed with PBS, and collected by centrifuging at 600 g, 4 °C for 5 min. Following the Caspase 3/9/8 Activity Assay Kit (Beyotime Biotechnology, Shanghai, China) instructions, the cells were resuspended in the lysis buffer at a ratio of 100 μL per 2 million, and incubated on ice for 15 min. Subsequently, they were centrifuged at 12,000 rpm for 15 min at 4 °C. The Caspase 3, Caspase 9, and Caspase 8 activities in cell lysates were determined using Caspase 3, Caspase 9 and Caspase 8 Assay Kits (Beyotime Biotechnology, Shanghai, China). The substrate for Caspase 3/9/8 was Ac-DEVD-pNA, Ac-LEHD-pNA, and Ac-IETD-pNA, respectively, and the concentration of all three substrates was 2 mM. The cells were incubated with the corresponding substrate and lysis buffer for 120 min. Substrate hydrolysis was assessed by measuring the absorbance change at 405 nm using a Multi-Mode Microplate Reader (BioTek, Vineland, NJ, USA). The enzymatic activity was quantified in arbitrary units (optical density, OD) per mg of protein.

### 4.10. Statistical Analysis

We performed each experiment independently three times. Statistical analysis was performed by using SPSS Statistics 27. Tests for normality and homogeneity of variance showed that our data were normally distributed and were of equal variance. All data are expressed as the mean ± SD (standard deviation). Differences between treated samples and control samples were analyzed by Student’s *t*-test. For multiple comparisons, the false discovery rate (FDR) was controlled below 0.05. The corrected *p*-values were calculated. The reported *p*-values in our manuscript are all the corrected *p*-values. A corrected *p*-value < 0.05 is considered statistically significant.

## 5. Conclusions

**Figure 8:**
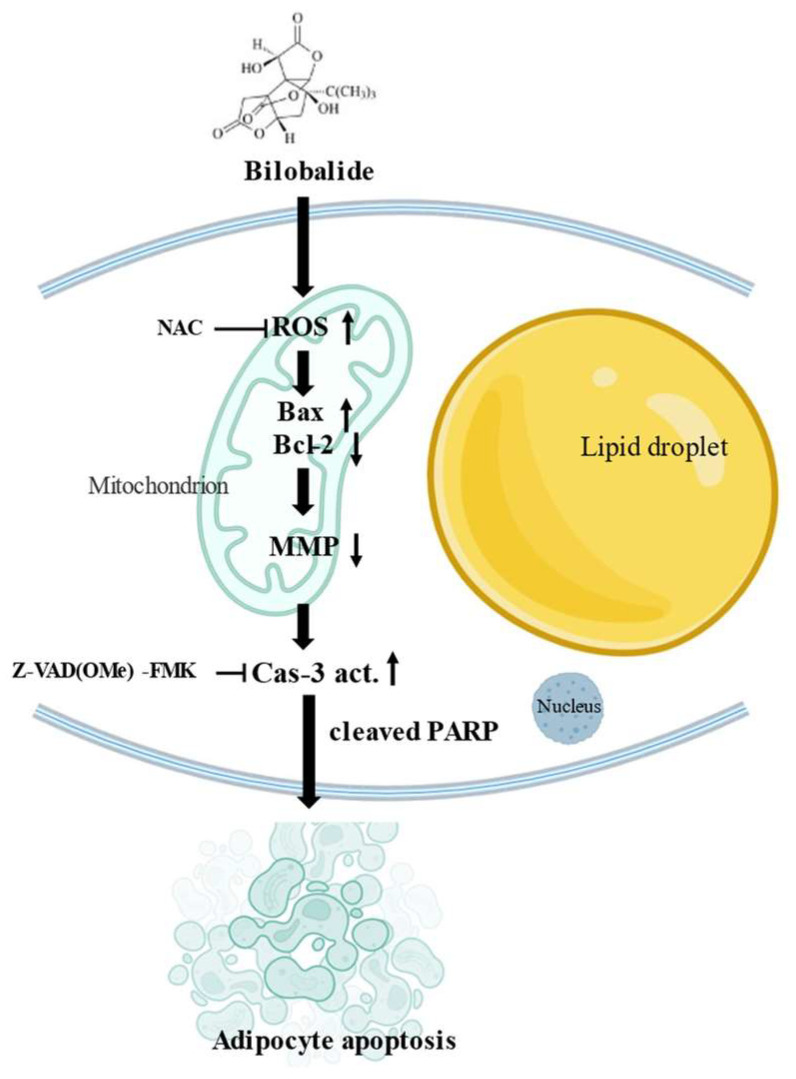
Schematic diagram of bilobalide-mediated apoptosis in 3T3-L1 adipocytes.

This study demonstrated that bilobalide can induce apoptosis in mature 3T3-L1 adipocyte through the ROS-mediated mitochondria pathway. FACS experiments demonstrated that bilobalide has a significant early apoptotic effect on mature 3T3-L1 adipocytes, significantly reducing the mitochondrial membrane potential in mature 3T3-L1 adipocytes. Moreover, bilobalide increased intracellular ROS levels and the activity of Caspase 3 and Caspase 9. The apoptotic effect of bilobalide on mature 3T3-L1 adipocytes was mediated by ROS. qRT-PCR and Western blot analyses of apoptosis-related proteins demonstrated a significant increase in the transcriptional expression of *Bax* (*p* < 0.001), and Western blotting revealed a significant increase in the Bax/Bcl-2, cleaved PARP/PARP, and cleaved Caspase 3/Caspase 3 ratio. The schematic diagram of bilobalide-mediated apoptosis in 3T3-L1 adipocytes is shown in Figure 8.

One advantage of bilobalide as a natural phytochemical is its relative safety compared to other synthetic weight-loss drugs available in the market [15]. However, further in vivo experiments, including optimal dosages and potential adverse reactions, are necessary to confirm the safety and efficacy of bilobalide as a weight-loss agent.

## Figures and Tables

**Table 1:** Primers used in this study.

Name	Primer Sequence (5′–3′)	GenBank No.	Size
*Bax*	5‘-TGCTAGCAAACTGGTGCTCA-3′	XM_011250780.3	183
	5′-GGCCTTCCTAATGCCAACCTG-3′		
*Bcl-2*	5’-CTTTGAGTTCGGTGGGGTCA-3’	NM_177410.3	168
	5’-GCCCAGACTCATTCAACCAGA-3’		
GAPDH	5’-TTTGTGGTGGGTGTGAACCA-3’	XM_029535298.1	469
	5’-TGAAGTCGCAGGAGACAACC-3’		

## Data Availability

Not applicable.

## Supplementary Materials

The following supporting information can be downloaded at: https://www.mdpi.com/article/10.3390/molecules28176410/s1.
