# Supplementary material for: Bilobalide Induces Apoptosis in 3T3-L1 Mature Adipocytes through ROS-Mediated Mitochondria Pathway
Source: Molecules. 2023 Sep 2;28(17):6410. doi: 10.3390/molecules28176410 (PMC10489643; doi:10.3390/molecules28176410)
Supplement: Supplementary file 1 [file molecules-28-06410-s001.zip › molecules-2528650-supplementary.pdf]

1.Marker
2.Cont 1
3.Cont 2
4.BB 25 μM-1
5.BB 25 μM-2
6.BB 50 μM-1
7.BB 50 μM-2
8.BB 100 μM-1
9.BB 100 μM-2

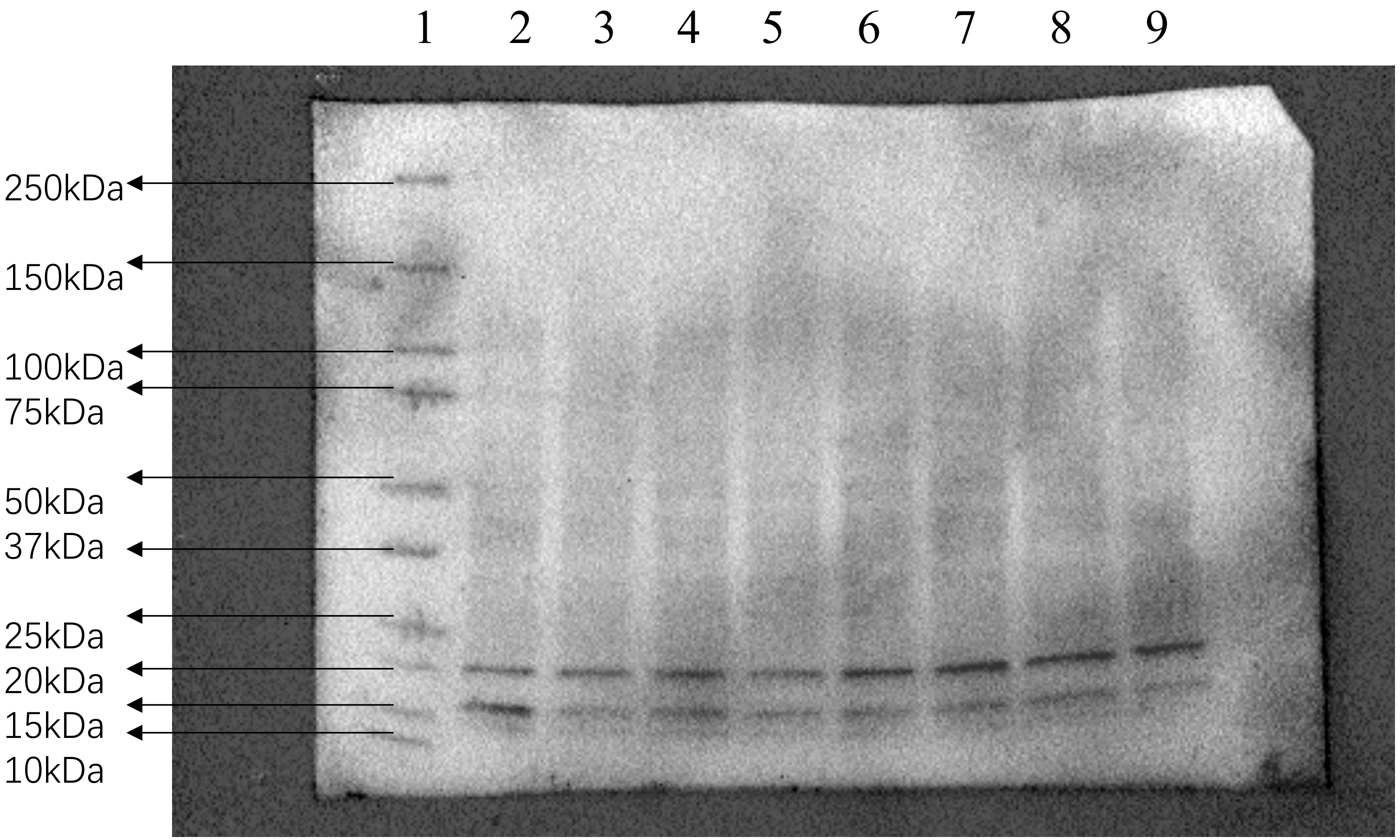

Bax 20 kDa

1.Marker
2.Cont 1
3.Cont 2
4.BB 25 μM-1
5.BB 25 μM-2
6.BB 50 μM-1
7.BB 50 μM-2
8.BB 100 μM-1
9.BB 100 μM-2

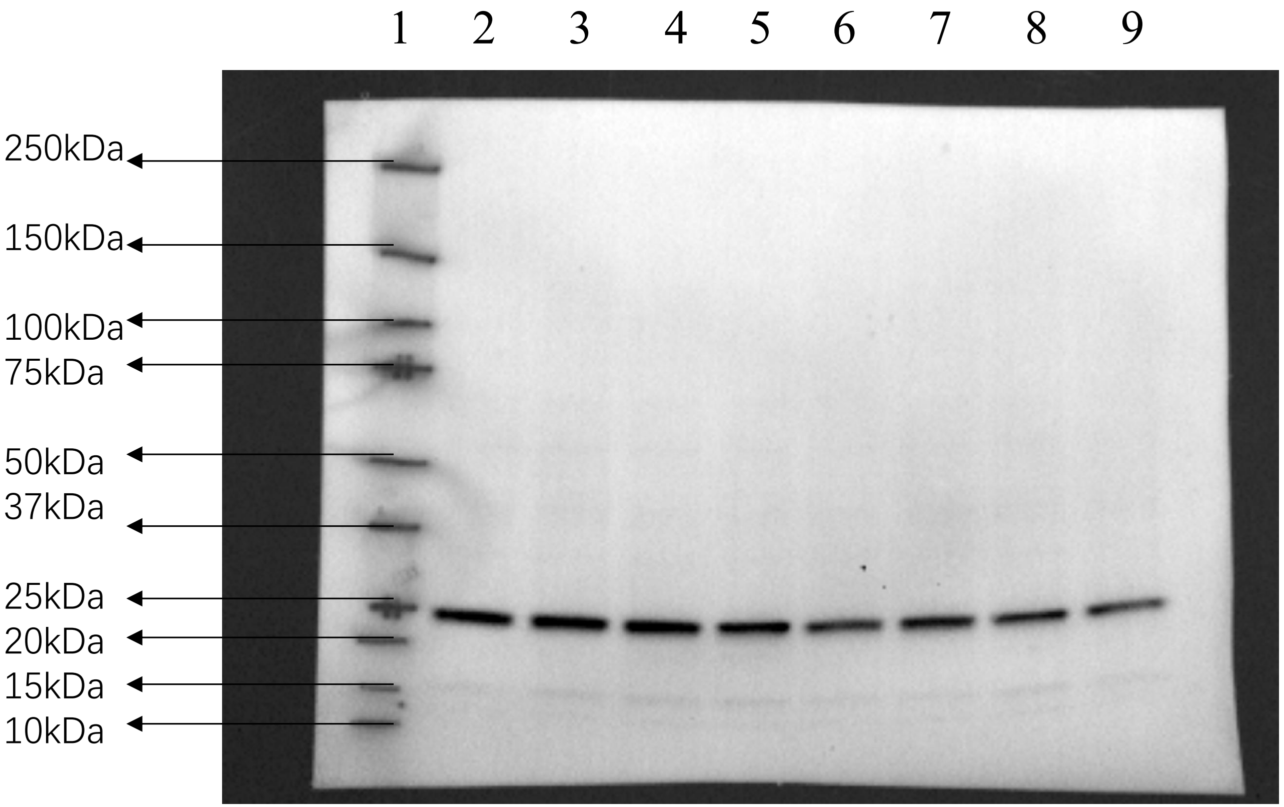

50 S

Bcl-2 26 kDa

1.Marker
2.Cont 1
3.Cont 2
4.BB 25 μM-1
5.BB 25 μM-2
6.BB 50 μM-1
7.BB 50 μM-2
8.BB 100 μM-1
9.BB 100 μM-2

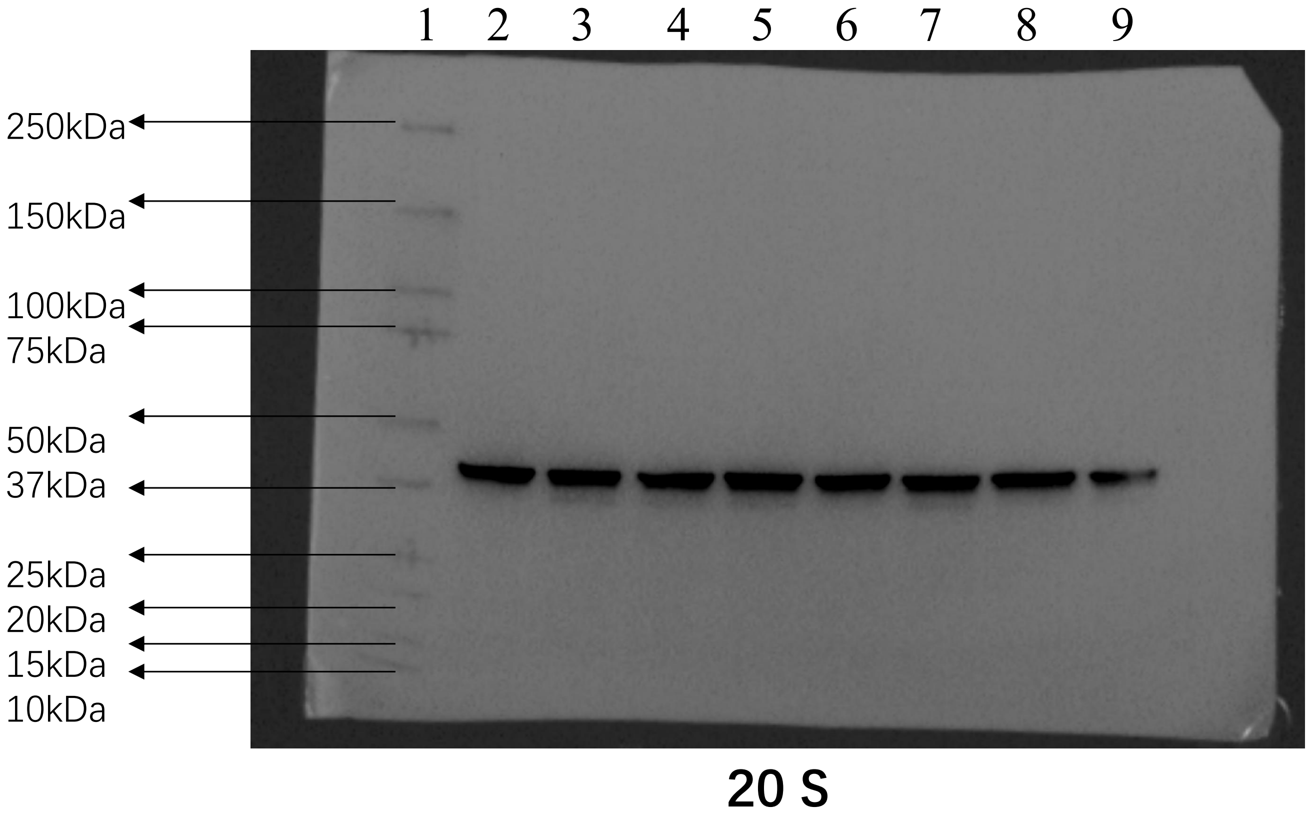

β-Actin 43 kDa

1.Marker
2.Cont 1
3.Cont 2
4.BB 25 μM-1
5.BB 25 μM-2
6.BB 50 μM-1
7.BB 50 μM-2
8.BB 100 μM-1
9.BB 100 μM-2

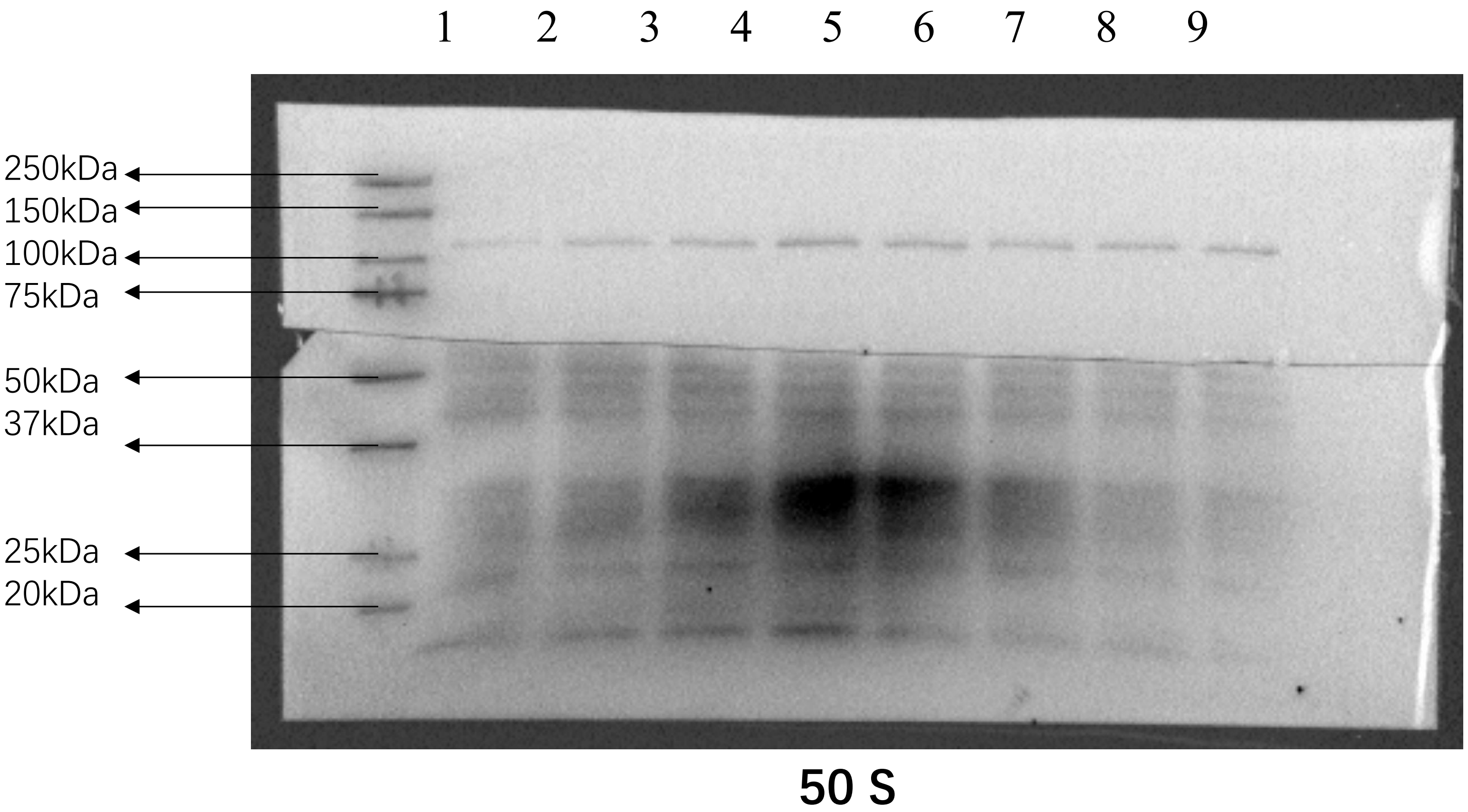

PARP 116 kDa
cleaved PARP 23 kDa

1.Marker
2.Cont 1
3.Cont 2
4.BB 25 μM-1
5.BB 25 μM-2
6.BB 50 μM-1
7.BB 50 μM-2
8.BB 100 μM-1
9.BB 100 μM-2

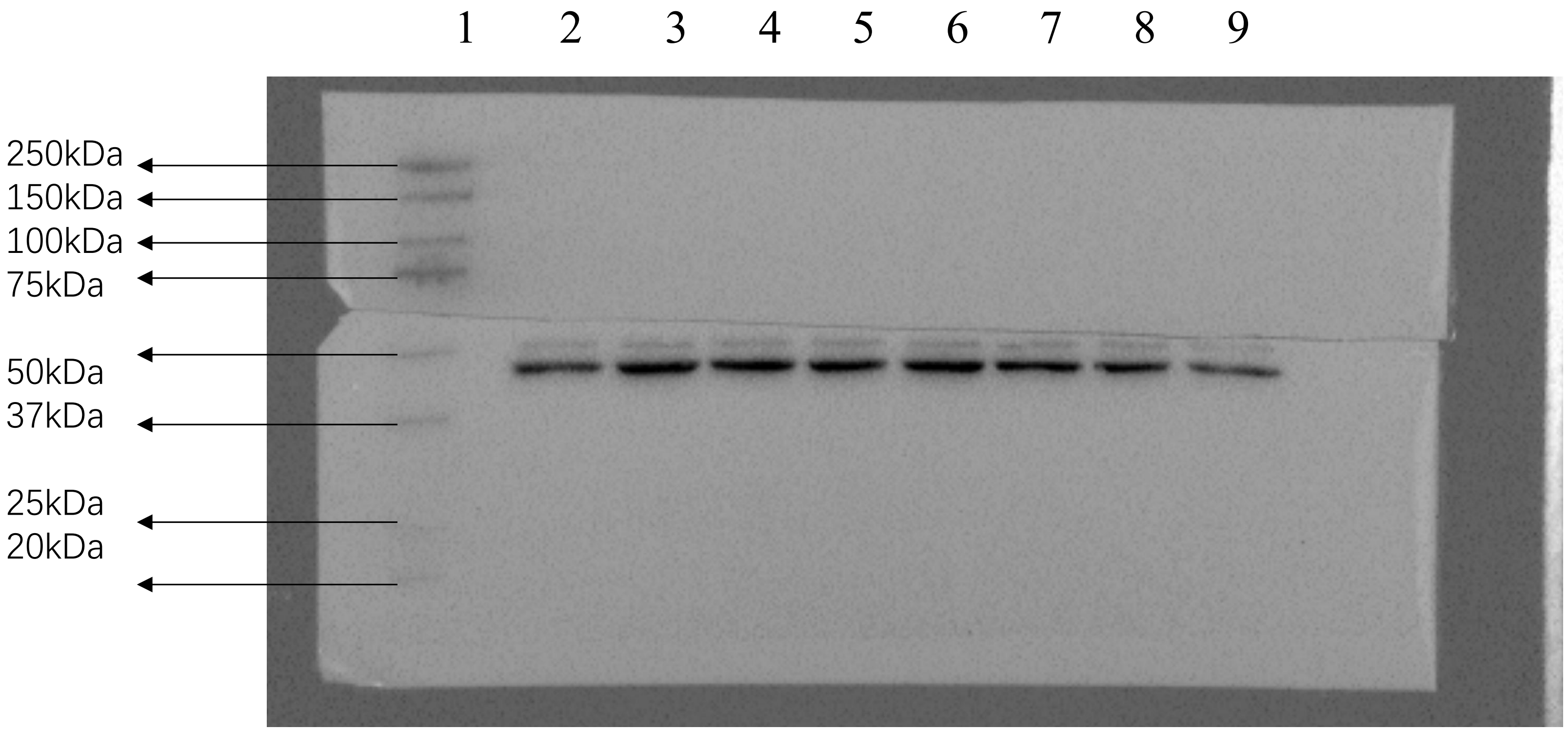

30 S

β-Actin 43 kDa

1.Marker
2.Cont 1
3.Cont 2
4.BB 25 μM-1
5.BB 25 μM-2
6.BB 50 μM-1
7.BB 50 μM-2
8.BB 100 μM-1
9.BB 100 μM-2

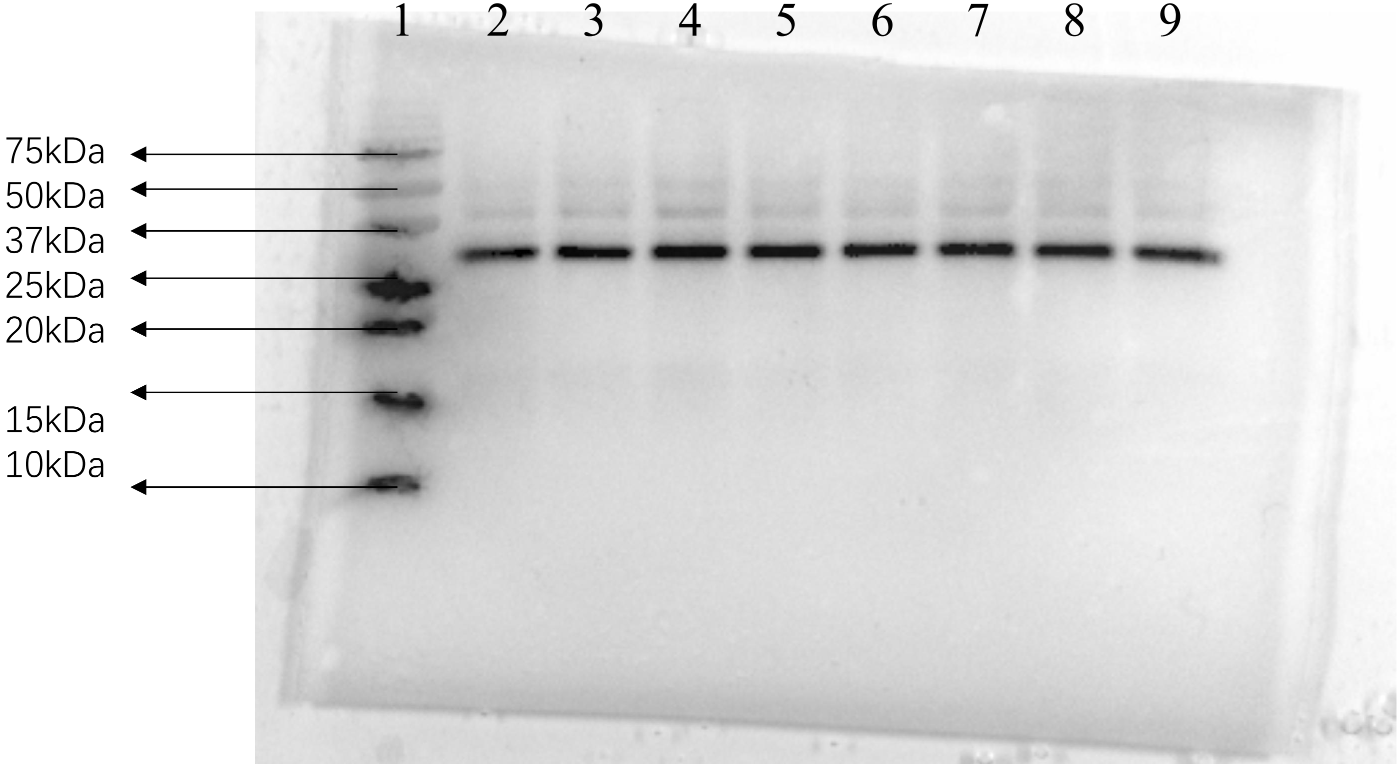

4.3 S

Caspase 3 35 kDa

1.Marker
2.Cont 1
3.Cont 2
4.BB 25 μM-1
5.BB 25 μM-2
6.BB 50 μM-1
7.BB 50 μM-2
8.BB 100 μM-1
9.BB 100 μM-2

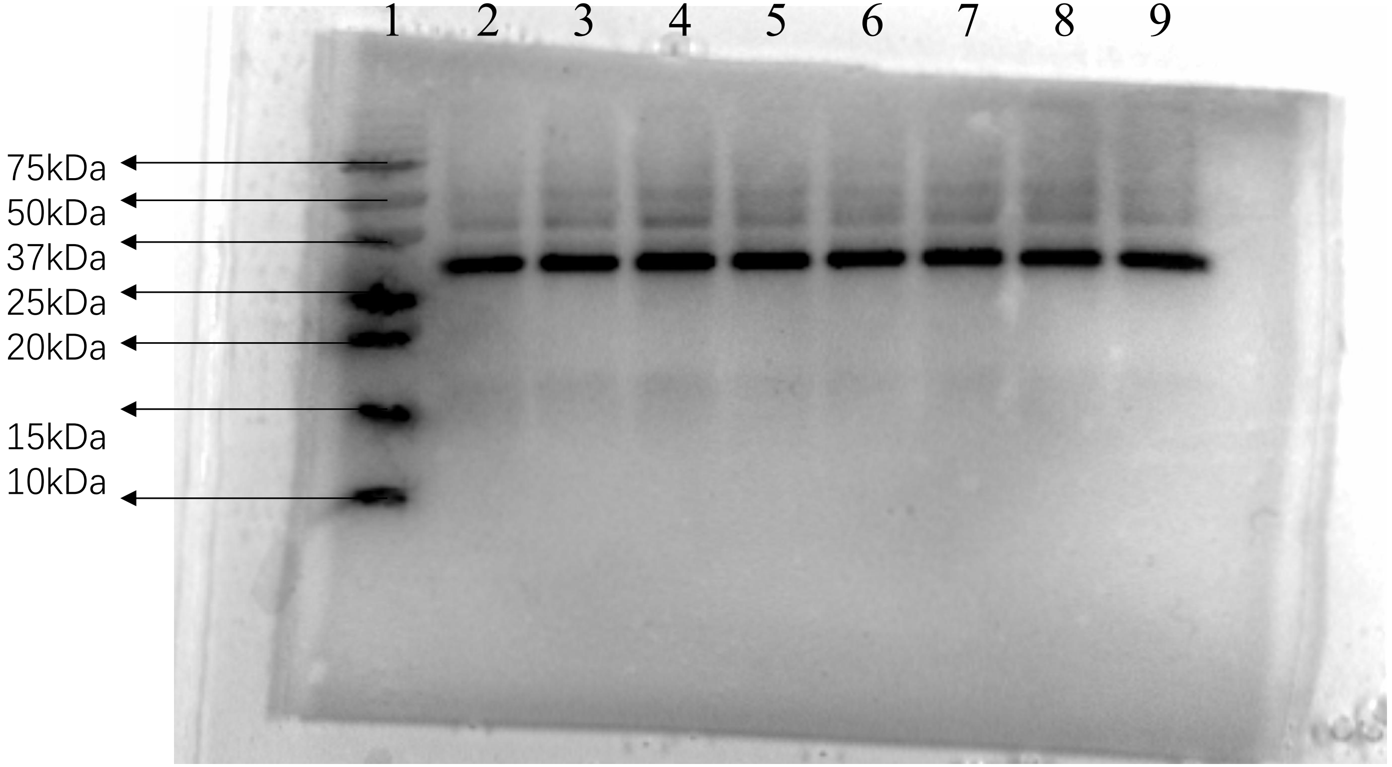

6 S

cleaved Caspase 3 19 kDa

1.Marker
2.Cont 1
3.Cont 2
4.BB 25 μM-1
5.BB 25 μM-2
6.BB 50 μM-1
7.BB 50 μM-2
8.BB 100 μM-1
9.BB 100 μM-2

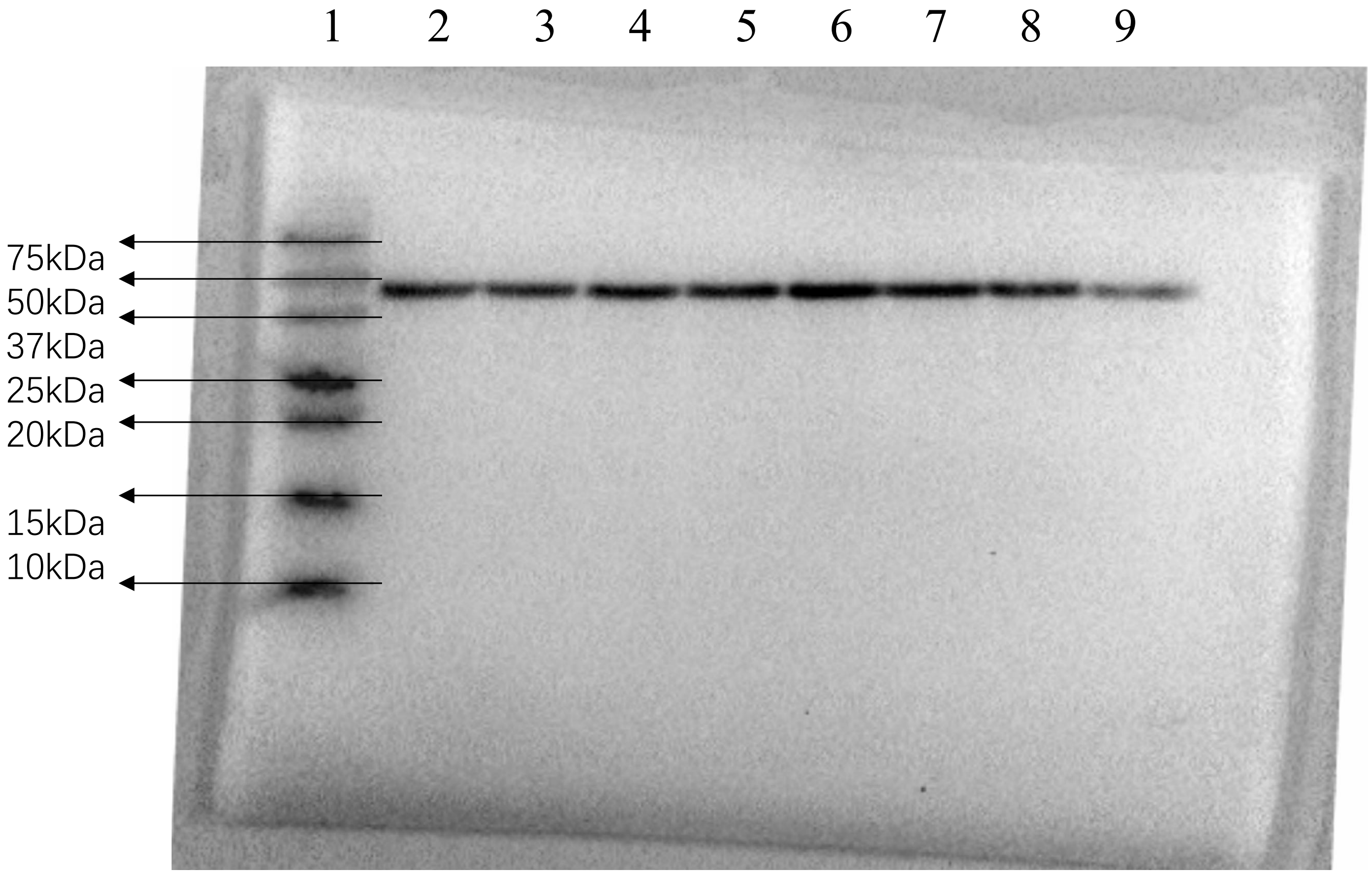

60 S

β-Actin 43 kDa

1.Marker
2.Cont 1
3.Cont 2
4.BB 25 μM-1
5.BB 25 μM-2
6.BB 50 μM-1
7.BB 50 μM-2
8.BB 100 μM-1
9.BB 100 μM-2

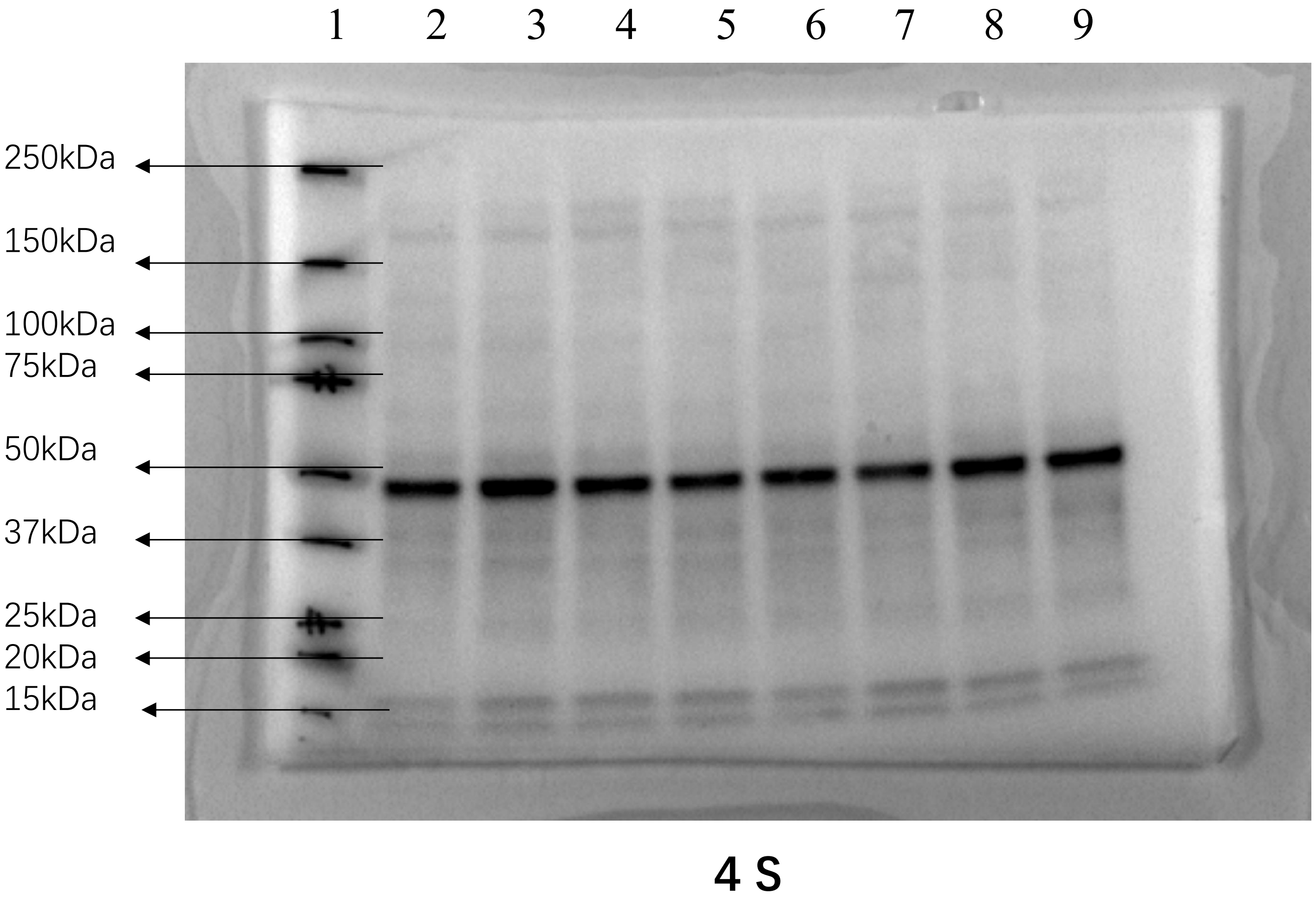

Caspase 9 46 kDa

1.Marker
2.Cont 1
3.Cont 2
4.BB 25 μM-1
5.BB 25 μM-2
6.BB 50 μM-1
7.BB 50 μM-2
8.BB 100 μM-1
9.BB 100 μM-2

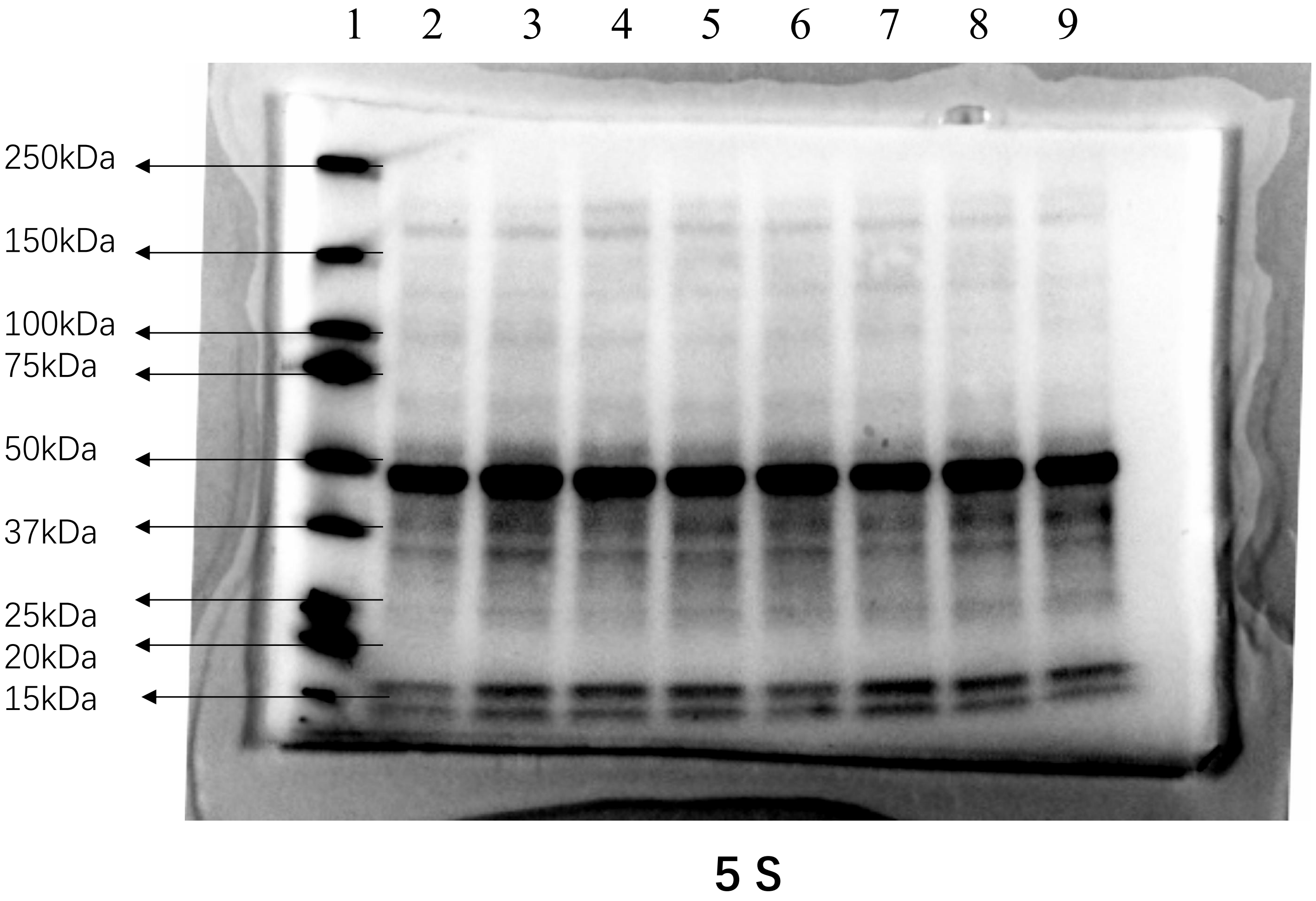

cleaved Caspase 9 39、37 kDa

1.Marker
2.Cont 1
3.Cont 2
4.BB 25 μM-1
5.BB 25 μM-2
6.BB 50 μM-1
7.BB 50 μM-2
8.BB 100 μM-1
9.BB 100 μM-2

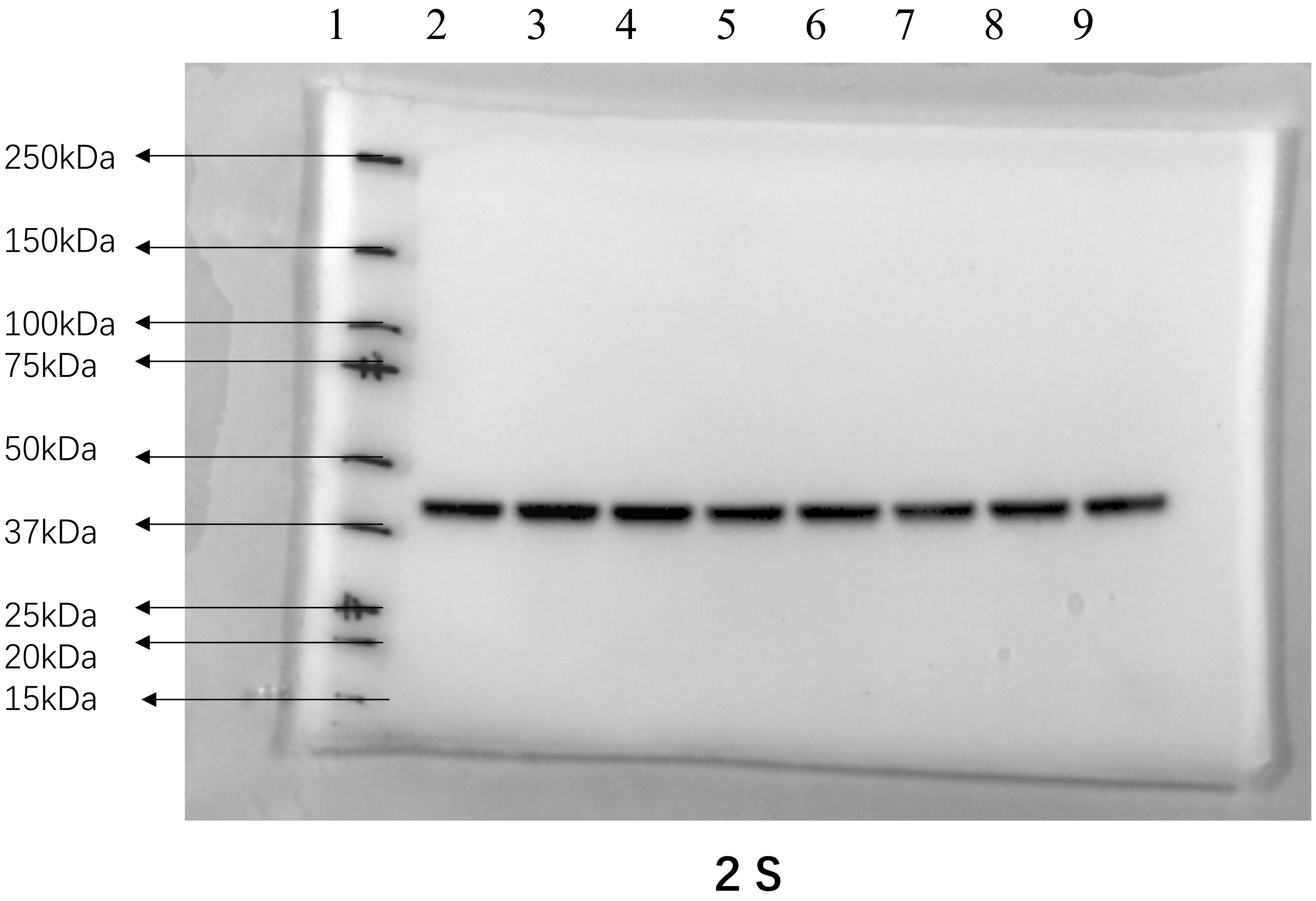

β-Actin 43 kDa

1.Marker
2.Cont 1
3.Cont 2
4.BB 25 μM-1
5.BB 25 μM-2
6.BB 50 μM-1
7.BB 50 μM-2
8.BB 100 μM-1
9.BB 100 μM-2

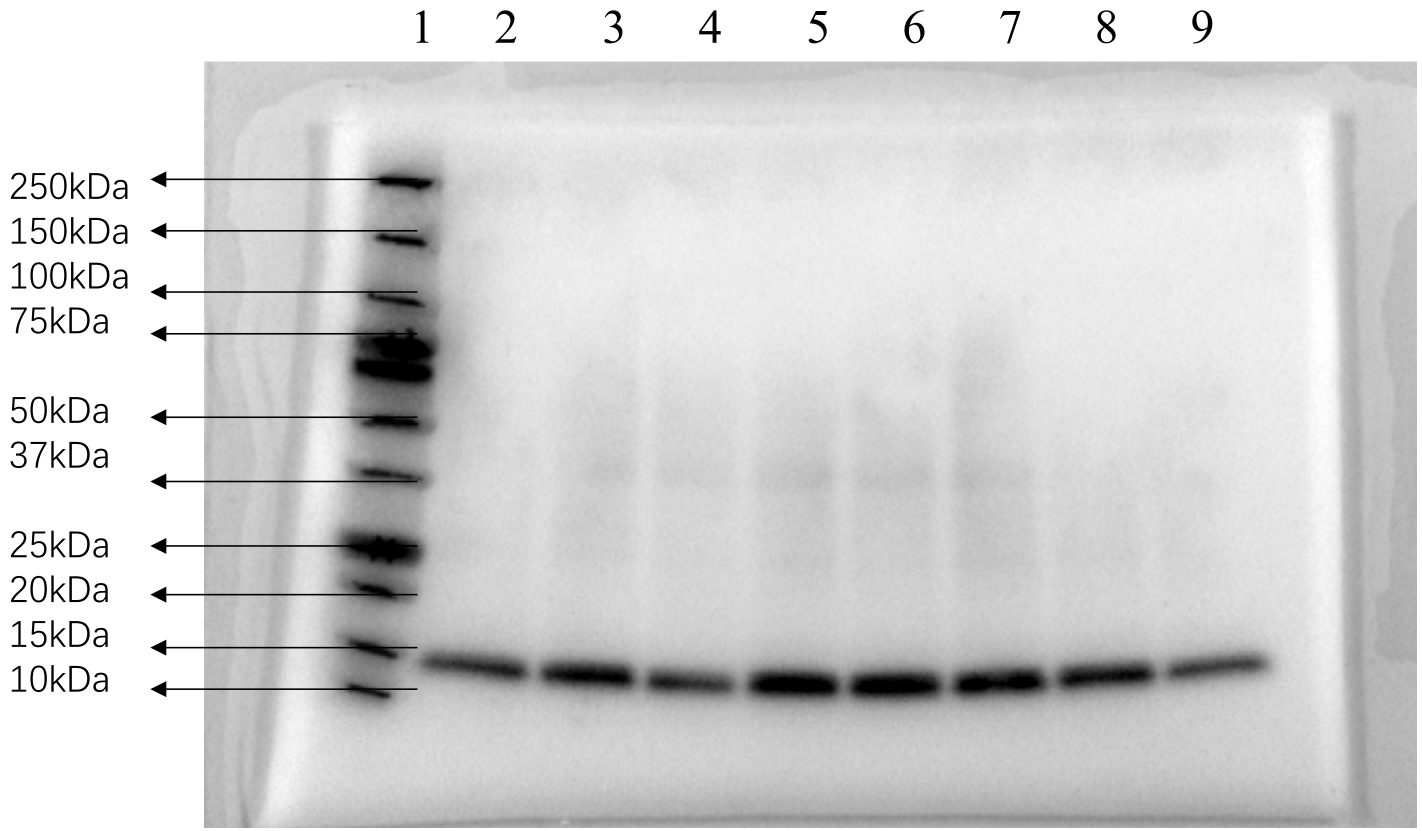

5 S

Cytochrome C 12 kDa

1.Marker
2.Cont 1
3.Cont 2
4.BB 25 μM-1
5.BB 25 μM-2
6.BB 50 μM-1
7.BB 50 μM-2
8.BB 100 μM-1
9.BB 100 μM-2

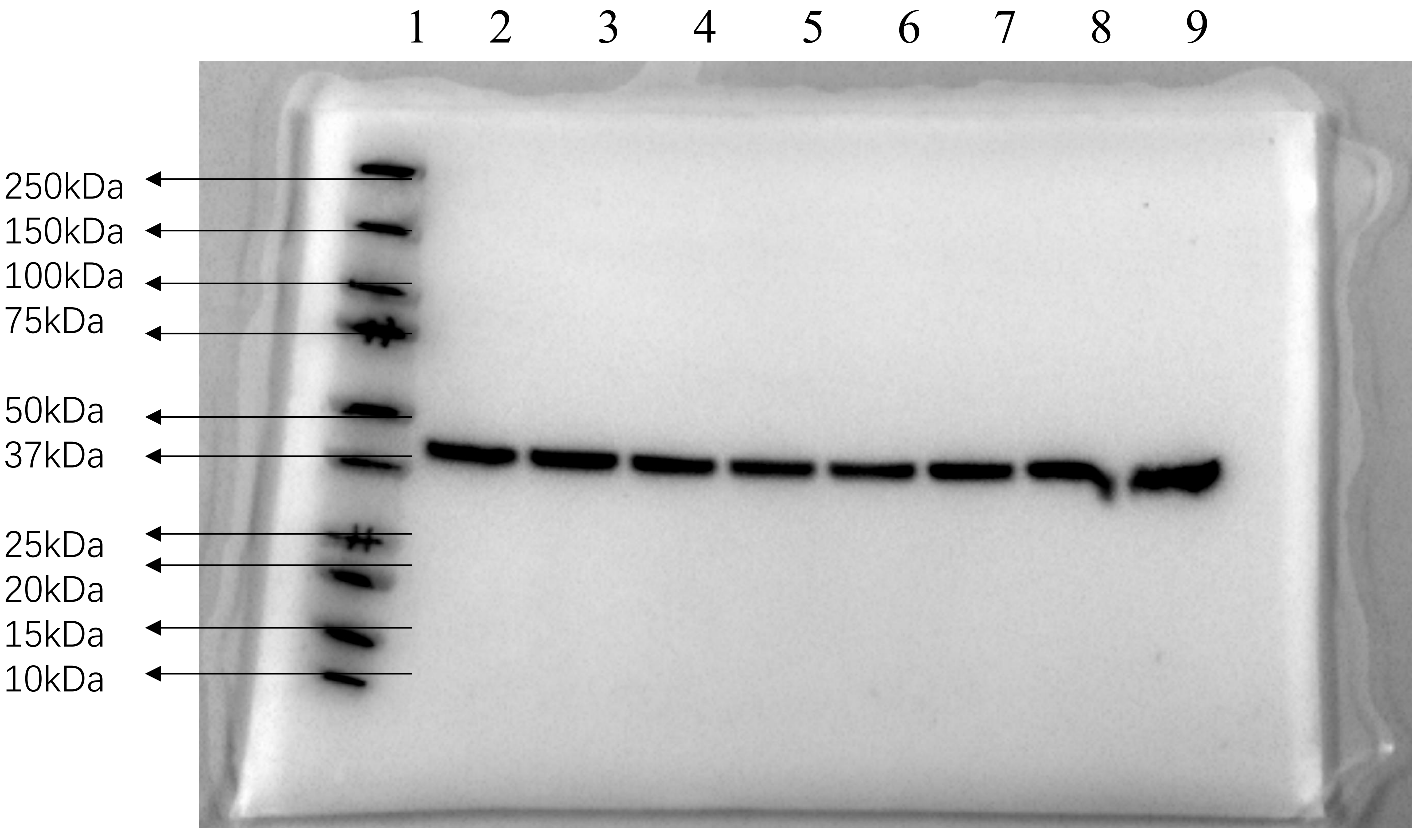

5 S

β-Actin 43 kDa
